# Mechanical Cell–Cell Communication in Fibrous Networks: The Importance of Network Geometry

**DOI:** 10.1007/s11538-016-0242-5

**Published:** 2017-01-27

**Authors:** D. L. Humphries, J. A. Grogan, E. A. Gaffney

**Affiliations:** Wolfson Centre for Mathematical Biology, Mathematical Institute, University of Oxford, Andrew Wiles Building, Radcliffe Observatory Quarter, Woodstock Road, Oxford, OX2 6GG UK

**Keywords:** Biopolymers, Discrete networks, Cell communication, Geometry

## Abstract

Cells contracting in extracellular matrix (ECM) can transmit stress over long distances, communicating their position and orientation to cells many tens of micrometres away. Such phenomena are not observed when cells are seeded on substrates with linear elastic properties, such as polyacrylamide (PA) gel. The ability for fibrous substrates to support far reaching stress and strain fields has implications for many physiological processes, while the mechanical properties of ECM are central to several pathological processes, including tumour invasion and fibrosis. Theoretical models have investigated the properties of ECM in a variety of network geometries. However, the effects of network architecture on mechanical cell–cell communication have received little attention. This work investigates the effects of geometry on network mechanics, and thus the ability for cells to communicate mechanically through different networks. Cell-derived displacement fields are quantified for various network geometries while controlling for network topology, cross-link density and micromechanical properties. We find that the heterogeneity of response, fibre alignment, and substrate displacement fields are sensitive to network choice. Further, we show that certain geometries support mechanical communication over longer distances than others. As such, we predict that the choice of network geometry is important in fundamental modelling of cell–cell interactions in fibrous substrates, as well as in experimental settings, where mechanical signalling at the cellular scale plays an important role. This work thus informs the construction of theoretical models for substrate mechanics and experimental explorations of mechanical cell–cell communication.

## Introduction

How cells interact with their substrate is of fundamental importance to many physiological processes, ranging from cell communication (Reinhart-King et al. 2008; Winer et al. 2009), motility and migration (Lo et al. 2000), cell fate (Engler et al. 2006; Gilbert et al. 2010) and morphology (Yeung et al. 2005). While changes in ECM may be signatures of pathology, including tumour invasion (Provenzano et al. 2006) and fibrosis (Wells 2005), it is increasingly clear that the passive mechanical properties of matrices are important for intrinsic cell function (Discher et al. 2005; Peyton et al. 2007). The major load-bearing structure in animal matrix is a fibrous network that is comprised of collagen, elastin and other proteins, which exhibits a geometrically complex and hierarchical structure. A clear understanding of this microenvironment is challenging, in part due to the non-affine response of the substrate (Wilhelm and Frey 2003; Chandran and Barocas 2006). Further complexity is introduced in cell-matrix interactions, which occur in a feedback loop termed ‘dynamic reciprocity’. Substrate reorganises under cell-generated tractions, leading to a strain-stiffening response which feeds back into an intracellular response. In particular, realignment of network fibres under traction leads to fibre bundling along the load direction, contributing to a stiffer mechanical response. Stress fibres, actomyosin bundles important for cell contraction, are subsequently recruited to sites of high stiffness, leading to further cell contraction, whence the feedback loop continues (Discher et al. 2005). Investigating how matrix alignment and stiffness are related is important for developing a better understanding of cell-matrix interactions.

Substrates derived from matrix proteins have diverse mechanical properties, contributing to their function. Perhaps, most striking is the ability to support long-ranging, cell-generated strain fields. In a series of experiments, Winer et al. (2009) demonstrated that fibroblasts and human mesenchymal stem cells (hMSCs) could significantly deform substrate more than five cell diameters away. This effect was notably absent in cells seeded on linear elastic substrates, such as polyacrylamide (PA) gels. Cell shape was acutely affected, with the round morphology displayed on linearly elastic substrates changing to larger spread area and greater elongation on strain-stiffening gels, similar to other investigations into cell morphology (Yeung et al. 2005).

It is now well established that cells respond to micromechanical cues within the substrate. Seminal experiments found that strong fibre alignment is induced between tissue explants seeded in collagen gels. These aligned fibres form tracts, which may function as topographic guides for cell migration (Stopak and Harris 1982). This phenomenon, termed ‘contact guidance’, is observed in tumour cell invasion, where cells migrate along aligned regions within ECM (Friedl et al. 1997; Jacques et al. 1998). Similar behaviour on the scale of individual cells has been identified in the formation of tethers, dense regions of highly aligned matrix fibres, between pairs of fibroblasts seeded on fibrin substrates (Notbohm et al. 2015). Cells have also been observed to migrate up stiffness gradients found within gels, a process known as ‘durotaxis’ (Lo et al. 2000; Plotnikov et al. 2012). Reinhart-King’s work, on cells seeded in compliant substrates (Reinhart-King et al. 2008), found that the motility of pairwise endothelial cells was dependent on both substrate stiffness and cell–cell interactions. Similar responses were absent on substrates that were either too soft, thus not supporting far reaching strain fields, or too stiff, in which cell tractions are unable to significantly deform the substrate. These studies provide evidence that mechanical cues, transmitted through the substrate, allow for pairwise cell interactions.

Various studies have also suggested that cells within matrix-derived gels can detect distant boundaries. While cells cultured upon PA gels can fail to sense substrate properties more than approximately $20\,{\upmu }$m beyond the cell membrane, hMSCs seeded on collagen-I gel appear to detect rigid boundaries further than ${100}\,\upmu \mathrm{m}$ away (Leong et al. 2010). Within a new model system, in which regular boundaries were introduced around cells on fibrous substrates, Mohammadi et al. (2014) found that cell-induced displacements extended beyond ${500}\,\upmu \mathrm{m}$, but failed to reach boundaries ${1700}\,\upmu \mathrm{m}$ distant (Mohammadi et al. 2014). Interestingly, when cells detected boundaries, they produced a greater number of protrusions, and these processes were longer on average, suggesting further physiological changes in cells introduced by the mechanical microenvironment.

Theoretical efforts have investigated phenomena such as nonlinear (strain-stiffening) matrix response and fibre realignment. With a strain-stiffening continuum model for the matrix, the relation between the far-field and local responses to active forces has been investigated, and scaling laws derived (Shokef and Safran 2012). Further, models in which the extracellular material stiffens under extension, and softens under compression due to fibre buckling, have shown that cell-derived traction forces may extend further than when such nonlinearities are omitted (Xu and Safran 2015). Other recent investigations have focussed on whole network models to represent the fibre matrix, in part due to the non-affine and heterogeneous response of fibrous substrates. Such models explicitly include fibres within a network structure, and break from affine assumptions found elsewhere in the literature (Heussinger et al. 2007). As the in vivo geometry and topology of matrices is not well established, it is often necessary to use either segmented imaging data, or to adopt an artificial geometry. Confocal microscopy data for a collagen-I network was used by Sander (2013) to propose a critical radius within which alignment of network fibres, due to cell contraction, is localised. Imaging of three-dimensional collagen networks allowed Stein et al. (2011) to confirm that geometric realignment, rather than filament-associated nonlinearity, could lead to the network strain-stiffening response. Cell communication was investigated through segmented images of fibroblast seeded collagen, demonstrating an increased range of stress transmission in fibrous substrates when compared with linear or strain-stiffening continua (Ma et al. 2013). Imaging data have the clear advantage that, in some sense, they represents the ground truth. However, in practice imaging artefacts are introduced, for example, through sample depth or misidentification of cross-links, entanglements or branching points. These artefacts limit the extent to which these networks may be taken as representative. Further, the difficulty of segmentation on the nano- and micro-scale limits the scope of using imaged networks. As such, there is great utility in the ability to generate representative artificial network architectures *in silico*.

While many models have employed artificially generated networks, the choice of network geometry remains relatively free (Picu 2011). Lattice models, such as triangular or Kagome, use regular, periodic geometries to represent the biopolymer network; such geometries may be diluted, say through bond deletion, until a desired density or persistence length is reached. Such models have recently been used to improve understanding of how contractile forces may be transmitted through 2 and 3D networks (Ronceray et al. 2016). Many models have also used less regular random structures, such as Mikado-type networks, generated by placing fibres of uniform length and random orientation within a two-dimensional domain (Head et al. 2003a, b; Onck et al. 2005). A cross-link is given by the intersection of two model fibres; as such network nodes have coordination *z* = 2, 3, or 4 once dangling ends have been removed. In a similar construction, presented by Chandran and Barocas (2006) and termed either ‘micromesh’ or ‘growth’ networks, uniformly seeded points are assigned an orientation, from which fibres grow until meeting another, whence a cross-link is formed and growth halts. Nodes in such networks have coordination *z* = 3. Voronoi-type geometries, which also have node coordination 3, have been used, where Voronoi edges are viewed as fibre segments, with vertices as cross-links. In two dimensions, Voronoi networks derived from random seed points also have coordination *z* = 3. If initial conditions are selected randomly, and a sufficiently large domain is chosen, these algorithms all produce isotropic networks.

The response of growth type networks was found to be non-affine, with little correlation between initial and final fibre orientation (Chandran and Barocas 2006). The ability for such networks to reorganise significantly reduced fibre strains. Growth geometries were used by Stylianopoulos and Barocas (2007b) to form a representative volume element (RVE). This RVE was used to motivate a macroscopic finite element model for collagen, finding good agreement with experiment. Investigations into polymer networks with a Mikado geometry have identified affine and non-affine regimes, the transition between which was described (Head et al. 2003a, b). More recent work involving Mikado-type networks underlined the importance of cell aspect ratio in long-range cell mechanical communication (Abhilash et al. 2014). Voronoi networks have been used to represent a discrete collagen scaffold, embedded within a continuous, neo-Hookean solid (Lake et al. 2012). This model resulted in good agreement with collagen gel data, and represented an holistic model, in which the fibrous network contributed to whole matrix mechanics. Other modelling approaches, alongside further details of those described above, are discussed in the thorough review by Broedersz and MacKintosh (2014).

While the above modelling efforts have developed the discrete fibre network into a powerful modelling tool, the freedom of choice in network geometry raises questions on the generality of their results. While it is known that prealigned geometries exhibited a far stiffer response at high strain (Lee et al. 2014), whether different isotropic networks behave differently is not addressed. We investigate the mechanical response of different isotropic networks that possess similar topology. We aim to quantify the significance of network architecture, comparing different geometries in a range of systems. In particular, we investigate the effects of network choice on the deformation field around a contractile cell, the degree of fibre reorganisation and alignment within the matrix, and the heterogeneity in mechanical response. We further investigate whether constituent fibre strain distributions are affected by geometry choice, and the plausibility of mechanical cell–cell communication within networks of different architectures. In summary, while controlling for material properties, together with network topology and cross-link density, we systematically investigate the relevance of geometry to the mechanical response of networks under uniaxial extension, local cell-derived tractions and cell–cell mechanical communication. These investigations are therefore likely to inform the theoretical modelling of substrate mechanics, as well as motivating experimental explorations into the micromechanical characteristics of fibrous substrates.

## Methods

### Model Fibre Choices

We present a whole network model for a biological matrix, in which fibres are included explicitly. Entropic effects are neglected, so that the model is athermal and purely mechanical. In particular, fibre segments are modelled as elastic springs, which form a network structure through point cross-links that are freely rotating. Fibres are assumed to have uniform diameter, so that the force-extension behaviour for each segment is equivalent. As such, our model takes the form of a central force network. Preliminary investigations, where deformations were induced by contracting cells, found that individual fibre strains were small, with very few fibres above 5% strain. Further, material nonlinearity emerges due to fibre network structure, rather than individual fibre properties (Stylianopoulos and Barocas 2007a). We therefore model fibres within the linear regime. To account for fibre buckling, a far softer fibre response in compression is used (Chandran and Barocas 2006), giving a piecewise linear force-extension law. The force *F* resulting from a fibre under extension or compression is therefore given by:

$$\begin{aligned} \qquad F \&= \ k \Delta L , \end{aligned}$$

where $\Delta L$ is difference between initial and final fibre lengths, $\Delta L = l - L$. The spring constant *k* is given through a representative Young’s modulus *E*, fibre cross-sectional area *A* and initial fibre length *L* as $k = EA/L$. A diameter of ${200}\,\mathrm{nm}$ was used as representative of a typical substrate fibre. A Young’s modulus $E_\mathrm{t} = {1}$ MPa was used for fibre extension. To represent the softer compressive or buckling response of fibres under compression, preliminary investigations were undertaken with Young’s moduli between 0.1 and 100  kPa. It was found that the mechanical network response was insensitive to this choice (results not shown), and for the results presented below a value of $E_\mathrm{c} = {10}$ kPa has been used for the Young’s modulus of a fibre under compression. The above modelling choices are similar to several existing studies in the literature (Chandran and Barocas 2006; Hudson et al. 2010; Lee et al. 2014; Notbohm et al. 2015).

Solutions are obtained through an overdamped, quasistatic scheme developed in the MATLAB (2014). Small fixed displacements are iteratively enforced at domain boundaries and interior nodes relax to effective equilibrium. In particular, for $\mathbf x ^n$, the position vector of the *n*th cross-link, we have :

$$\begin{aligned} \qquad \qquad \frac{\text {d}{} \mathbf x ^n}{\text {d}t}&= \frac{1}{\mu }\sum \limits _{i} k_i^n \left( l_i^n - L_i^n\right) \mathbf t _i^n \ , \end{aligned}$$

where summation is over nodal fibres, $l_i, L_i$ are current and initial (natural) fibre lengths and $\mathbf t _i$ gives the current fibre direction, $||\mathbf t _i^n|| = 1$. $k_i^n$ gives the relevant spring constant for extension or compression, and $\mu$ is a damping term. For numerical solutions, the above equation is non-dimensionalised with scales chosen such that there is unit damping. The overdamped system relaxes; numerically, the termination condition for this relaxation is given by the requirement that the maximum force on any network cross-link is six orders of magnitude smaller than the force scale associated with fibre extensions on the scale of the natural length, ensuring effective force balance. While units are given as representative of substrate fibres, we emphasise that these values are taken so as to facilitate a comparative study into different network geometries rather than relate to experiment. As such, results are insensitive to these parameter choices within the overdamped limit.

### Network Geometry

Network architecture is defined by both a graph structure, the network topology, and the network geometry, that is the spatial embedding of the graph. In order to investigate the impact of geometry on the network mechanical response, we select artificial networks of a given type. In two dimensions (2D), it is possible to select networks used in existing models which have distinct geometric properties, but whose topology is similar. Furthermore, many of the interesting properties of fibre networks, including strain-stiffening, non-affine response and fibre alignment are observed in both two and three dimensions (Onck et al. 2005; Hu et al. 2012). We therefore restrict ourselves to 2D networks, which will also be of greater relevance to the many 2D studies published to date.

Throughout, network nodes are viewed as permanent cross-links between two fibre segments. We investigate networks with node coordination $z = 3$, similar to typical values for fibrin networks (Notbohm et al. 2015). In particular, Voronoi- and ‘growth’- type geometries are studied. Voronoi networks are generated by seeding *n* random points uniformly in a given domain as generators, from which a Voronoi diagram is formed. The resulting Voronoi edges are viewed as fibre segments, between either fibre branching points or cross-links. For comparison, we also produce growth type networks, as outlined by Chandran and Barocas (2006). These networks are generated by again seeding *n* random points within the computational domain, and assigning each seed a random direction. Fibres extend forwards and backwards in this direction until contacting either another fibre segment or a domain boundary, whence a cross-link is formed and the growth terminates.

**Fig. 1:**
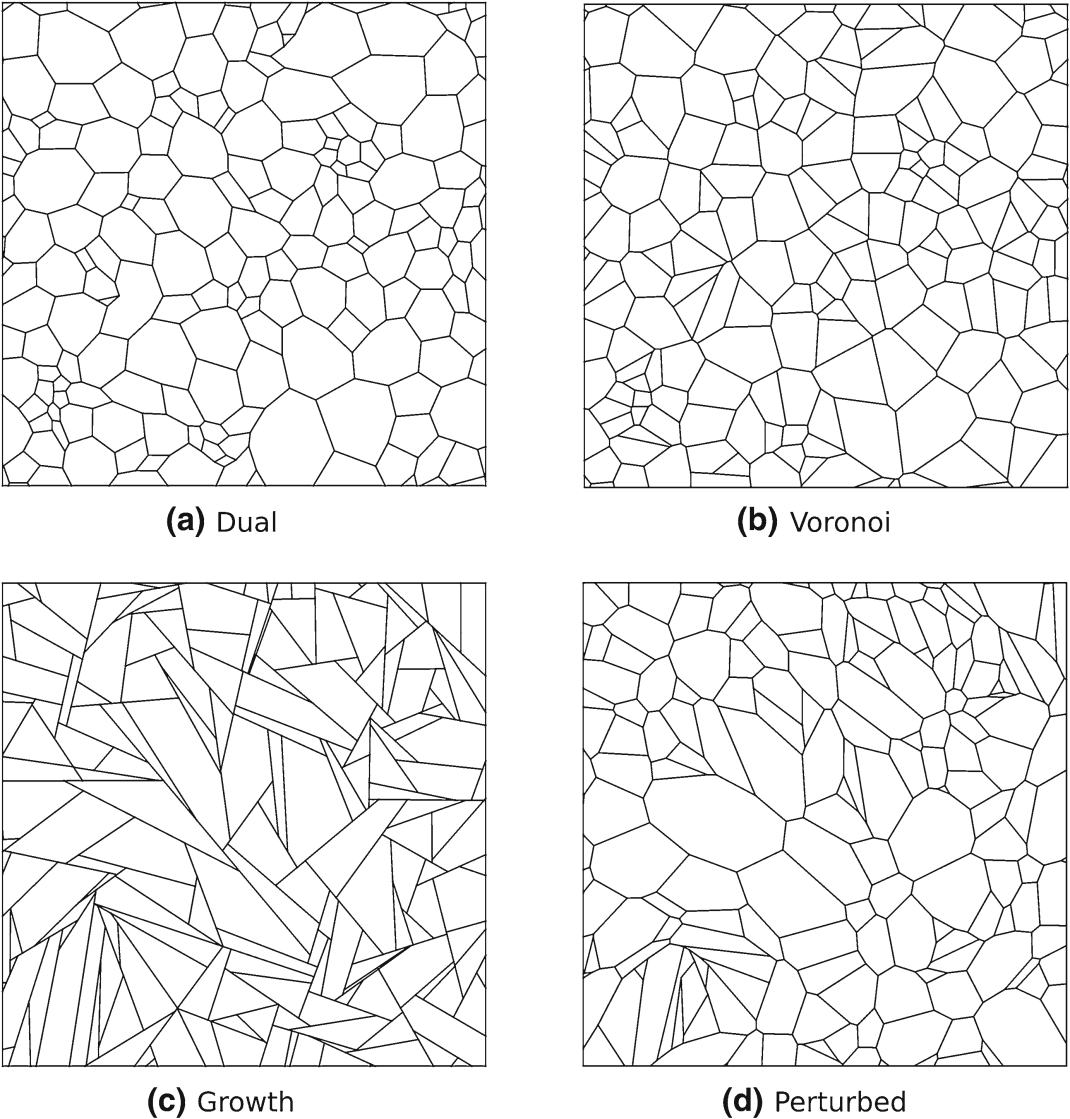
Visualisation of the local structure and geometry for each model architecture. Details for network generation are given in Sect. 2.2

Producing Voronoi networks from random seed points produces a random network with little local structure. In contrast, each interior node in growth geometries supports precisely two parallel edges, so that an angle of $\pi$ is present between two fibre segments at each node (see Fig. 1c). As we wish to investigate the importance of geometry, we control for network topology as follows. Two further network types, which can be viewed as geometric ‘perturbations’ of the two networks architectures considered, are introduced. Firstly, using the same Delaunay triangulation corresponding to a given Voronoi network, we produce a different dual graph. This is achieved by introducing nodes at each triangle centre, which are then connected to neighbouring centres through shared triangle edges. In this manner, a network with similar topology to the Voronoi case is generated. We refer to this geometry as ‘dual’- type throughout. A perturbation to growth networks is performed by introducing a small tension into each fibre segment, whence the system is allowed to relax to equilibrium. This equilibrium state is then taken as a strain-free network. In this way, the local structure observed at each node in growth networks, where each node shares two fibre segments separated by an angle of $\pi$, is removed. This perturbed network is thus a different spatial embedding of the same graph produced by growth networks, and differences in the mechanical response of these two network types derive purely from this geometric perturbation, rather than from the network topology. Throughout, these networks are referred to as ‘perturbed’-type. The Kolmogorov–Smirnov test (Massey Jr 1951) was used to confirm that all networks considered were nominally isotropic, in the sense that networks exhibited no preferential fibre orientation. All networks are considered initially stress free, so that initial and natural fibre lengths coincide. Visualisations of each model geometry are shown in Fig. 1.

As cross-link and fibre density cannot be independently specified for each geometry in 2D, we control for cross-link density, which has been found to strongly influence fibre network mechanics (Lin and Gu 2015). Except where otherwise specified, networks of cross-link density ${0.2}\,\upmu {\mathrm{m}}^2$ are used. This gives corresponding mean fibre densities of 0.63, 0.70, 0.8 and ${0.67}\,\upmu \mathrm{m}/ \upmu {\mathrm{m}}^2$ for dual, Voronoi, growth and perturbed networks respectively. We note the slightly lower fibre density for dual geometries and slightly higher density for growth networks, which is a consequence of fixing cross-link density.

**Fig. 2:**
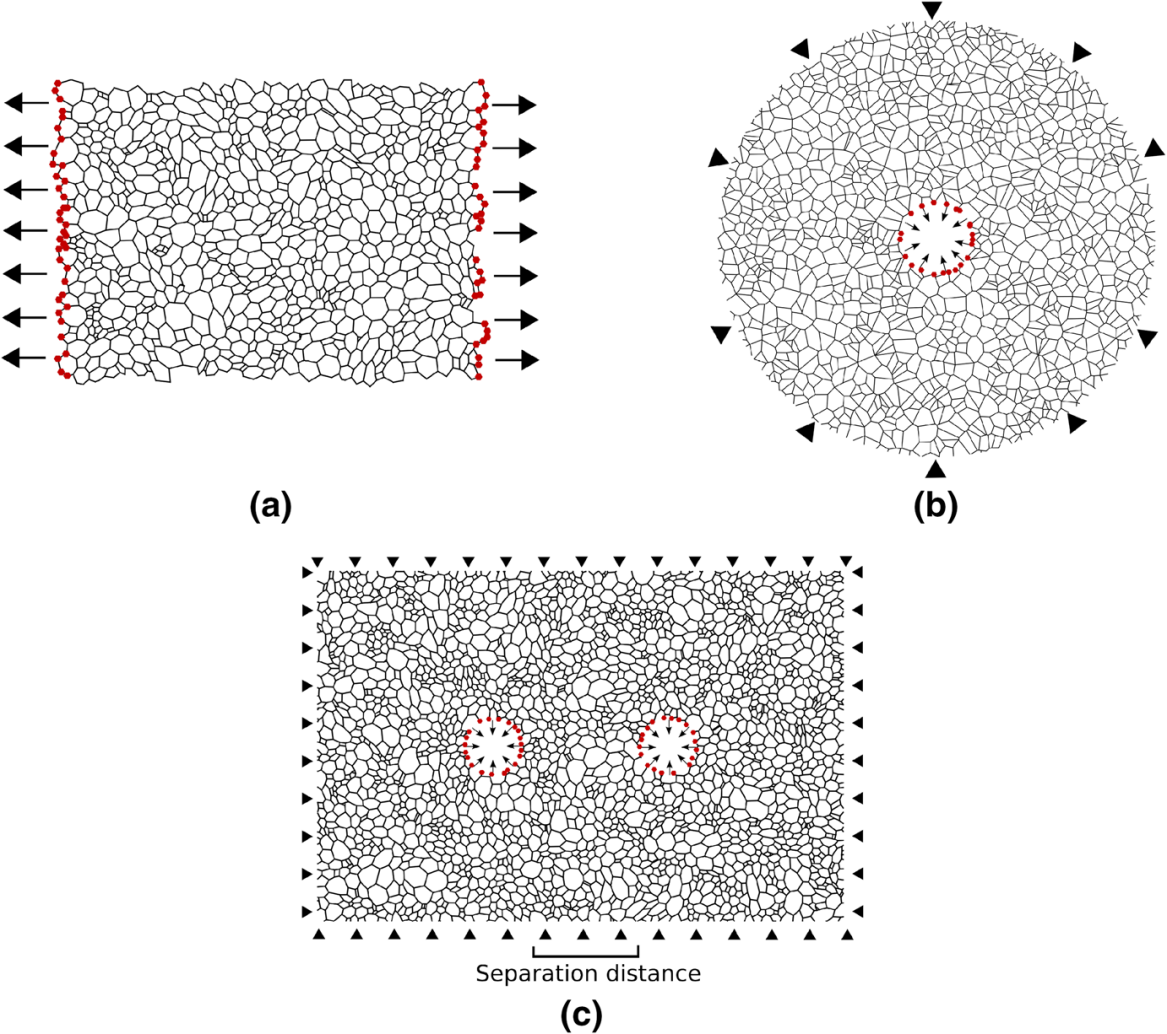
(Colour figure online) Model domains for computational tests. Displaced boundary nodes are highlighted in *red*. **a** Uniaxial extension of a dual type network. Boundary nodes are displaced *horizontally*. **b** Single cell contraction within a Voronoi network. An *interior circle* is removed from the domain, and inner boundary nodes are displaced in the radial direction towards the centre, with outer nodes fixed. **c** Pairwise mechanical cell communication within a dual network. Two *interior circles* are removed from a network within a rectangular domain. Cells contract as in the single cell case. The separation distance between pairwise cells is defined to be the shortest membrane–membrane distance. *Triangular markers* signify fixed boundary nodes

Three mechanical tests are employed to investigate the importance of network geometry at different scales. Firstly, networks are subjected to uniaxial extension, as in Fig. 2a. Boundaries are defined so as to include boundary polygons, thus avoiding free nodes. A domain size of approximately ${100}\,\upmu \mathrm{m}$ is selected to ensure an isotropic distribution in fibre orientation. Boundary nodes (marked in red, Fig. 2a) are subjected to fixed horizontal displacement. Single cell contractions are modelled by removing a circular region from a network, as in Fig. 2b. The inner circular region, representing a cell, is allowed to contract radially, while nodes attached to the outer boundary remain fixed. Model cells have initial diameter of ${20}\,\upmu \mathrm{m}$ and final diameter of ${10}\,\upmu \mathrm{m}$, within a domain of diameter ${120}\,\upmu \mathrm{m}$. Mechanical cell–cell communication is investigated by introducing cells (final diameter ${10}\,\upmu \mathrm{m}$) into a larger rectangular domain such that boundaries are never closer than in the single cellular case, as shown in Fig. 2c. A cell separation distance ranging between 50–70 $\upmu \mathrm{m}$, corresponding to separations of 5–7 final cell diameters, is used to investigate the ability for cells to communicate mechanically. All results are averaged across 20 network realisations, allowing for an investigation of network to network variability. Additional simulations on networks with more distant boundaries ensured that boundary effects did not significantly affect the results given.

In summary, we investigate the mechanics of four different network architectures. Each network is paired with another of similar or identical graph structure, to reduce the effects of topology. We control for density by fixing cross-linking density across all networks. Equivalent material parameters are used for all network choices. Mechanical tests are performed so as to investigate bulk and local responses of different network architectures. As such, this model is designed to isolate the importance of geometry to the mechanical response.

## Results

### Network Stiffness Under Uniaxial Extension is Dependent on Network Choice

**Fig. 3:**
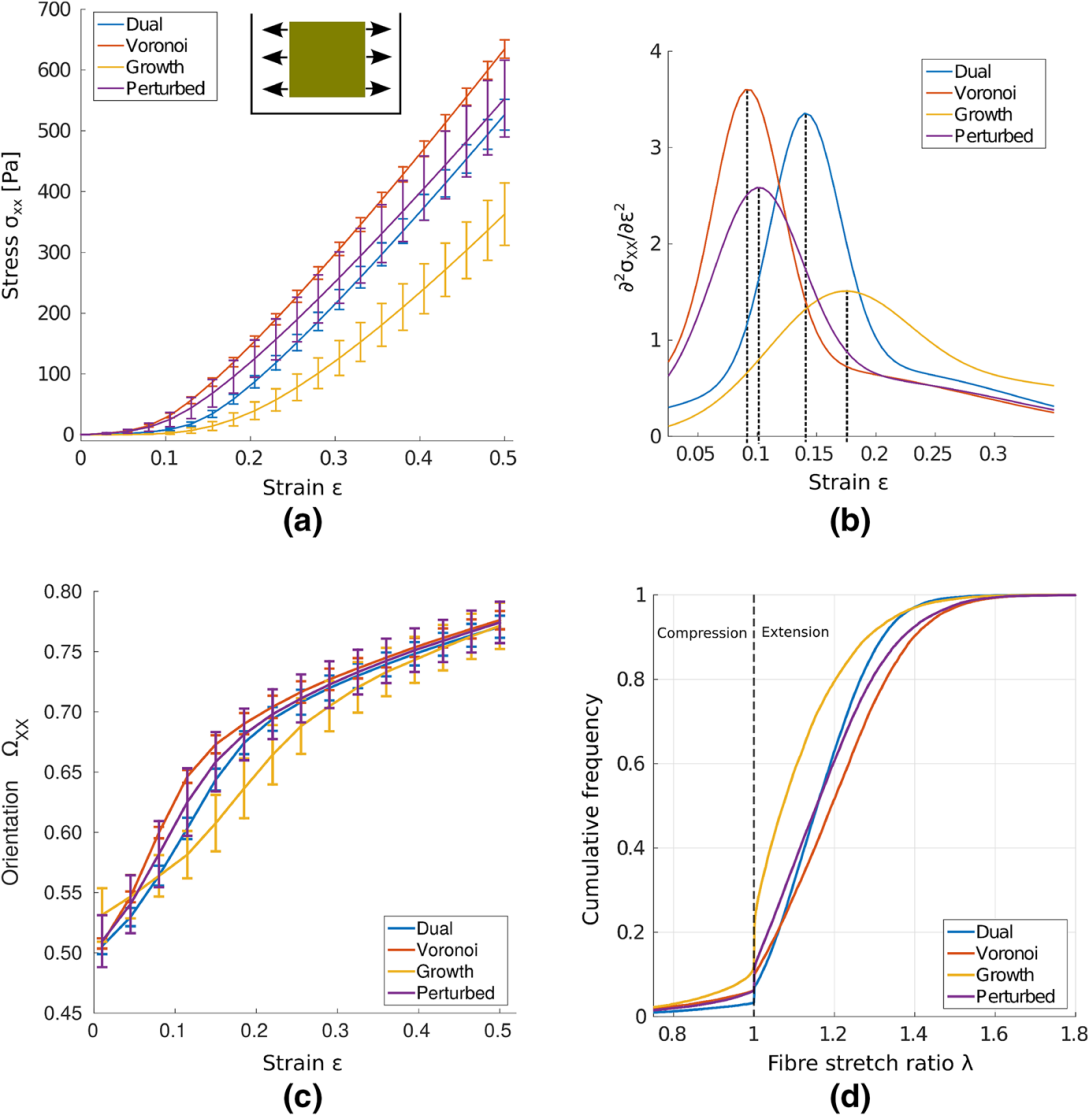
(Colour figure online) Mechanical response of networks under uniaxial extension. **a** Engineering stress–strain curves show that Voronoi networks are relatively stiff, while growth cases exhibit the softest response. The *inset* shows the uniaxial test performed. **b** The second derivative of stress with strain is plotted. All networks exhibit strain-stiffening, though the onset of strain-stiffening occurs later in dual and growth architectures. Voronoi and perturbed networks display similar strain-stiffening response. **c** The orientation parameter $\Omega _{XX}$ increases with strain for all networks, as constituent fibres reorient with the direction of extension. **d** Cumulative fibre stretch ($\lambda$) distributions within different network geometries at 50% strain underline micromechanical differences, with far fewer strained, and more buckled, fibres present in growth networks than in Voronoi geometries. *Bars* give 1 SD about the mean value

We first investigate the response of different networks under uniaxial extension, as shown schematically in Fig. 2a. Many substrate gels and ECM exhibit a strain-stiffening response, with characteristic toe, heel and linear regions (Fratzl 2008, chap. 17). Engineering stress–strain curves for each network considered are given in Fig. 3a. As strain increases to $50\%$, all networks exhibit a strain-stiffening response. Interestingly, a far stiffer response is present in Voronoi than growth networks, with stress at $50\%$ strain in Voronoi geometries almost double that found in the growth case. To investigate the qualitative difference in response, we compare the second derivative of stress with strain, that is a measure of strain-stiffening, in Fig. 3b. The onset of strain-stiffening in perturbed and Voronoi networks occurs around 10% strain, with a later response in dual networks, and later again for growth cases, above 15% strain. Given that fibres follow a piecewise linear force-extension curve, this nonlinear response is purely geometric, due to the reorganisation of network fibres. As such, the perturbation to the growth type geometry, has the effect of changing both the quantitative stiffness and the qualitative shape of the stress–strain curve under uniaxial extension, suggesting that geometry plays a key role in substrate network mechanics.

We investigate the cause of this increase in stiffness from a micromechanical perspective, looking at single fibre properties. Firstly, we investigate network fibre alignment. We introduce the orientation parameter:

1 $$\begin{aligned} \qquad \Omega _{ij} := \frac{\sum \nolimits _{k} p_i^k p_j^k}{\sum \nolimits _{k}|\mathbf p ^k|} \ , \end{aligned}$$

as a measure of geometric alignment. Here, $\mathbf p$ are vectors describing a given family of *k* network fibres, while *i*, *j* are axis directions. For alignment with the direction of extension that is with the horizontal, we have:

2 $$\begin{aligned} \qquad \Omega _{XX} := \frac{\sum \nolimits _{k}|\mathbf p ^k|\cos ^2\theta ^k }{\sum \nolimits _{k}|\mathbf p ^k|} \ , \end{aligned}$$

where $\theta ^k$ is the angle between fibre and the positive *x*-axis. A value of 0.5 represents nominal isotropy, while a value of 1 gives a perfectly aligned network. We expect fibres to buckle rather than compress, whence alignment ceases to be meaningful. We therefore neglect to include fibres with $\lambda < 1$, where stretch ratio $\lambda := l/L$ gives the ratio of final to initial fibre length. The change in orientation parameter as strain increases is shown in Fig. 3c, showing increasing alignment in all networks. Orientation response is similar in dual, perturbed and Voronoi cases, with significant differences in growth networks, as strain increases. However, significant reorientation of network fibres with the direction of loading is observed in all networks, with similar alignment from $30\%$ strain.

Individual fibre strain throughout the network is investigated for bulk uniaxial extension. Cumulative frequency distributions for fibre stretch are shown in Fig. 3d. Interestingly, growth type networks contain considerably more buckled, and fewer strained fibres than other network geometries. As buckled fibres are not included in our consideration of alignment, this provides a partial explanation for the different orientation response of growth networks. In contrast, Voronoi geometries favour a greater number of strained fibres.

Given the above differences in stiffness under a bulk mechanical test, we proceed to investigate whether these differences persist under local perturbations, and the implications for cell function in different network architectures.

### Displacement Fields Arising from Single Contractile Cells Exhibit Qualitative Differences with Choice of Initial Network Geometry

**Fig. 4:**
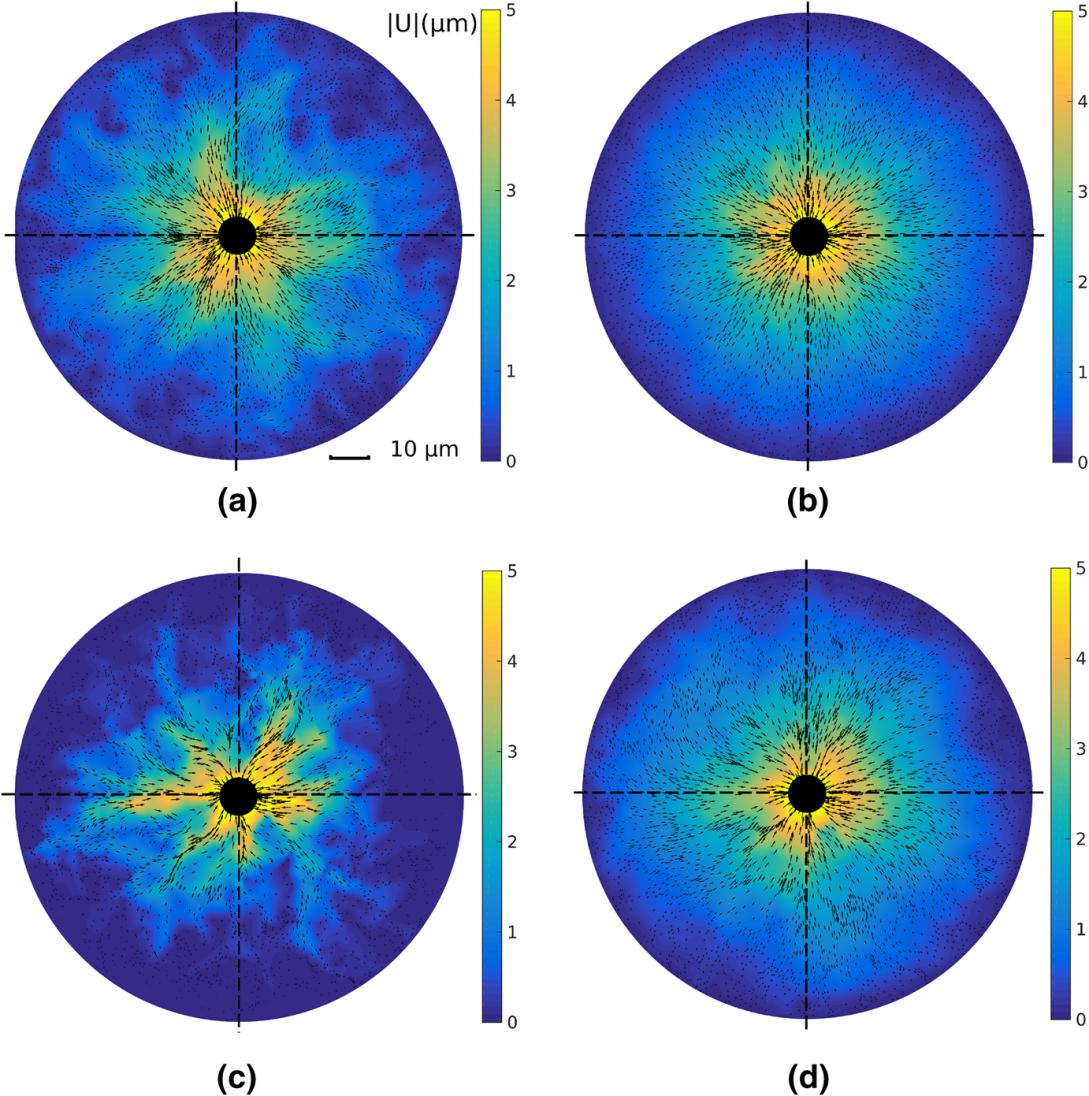
(Colour figure online) Displacement fields $|\text {U}|$ ($\upmu \mathrm{m}$) due to a contractile cell show strong qualitative differences as network architecture is changed. In particular, largely symmetric fields are observed in Voronoi geometries, while a highly heterogeneous field, involving symmetry breaking, results in growth networks. *Black arrows* give displacement vectors for fibre midpoints. Continuous plots here and throughout are generated through natural neighbour interpolation

**Fig. 5:**
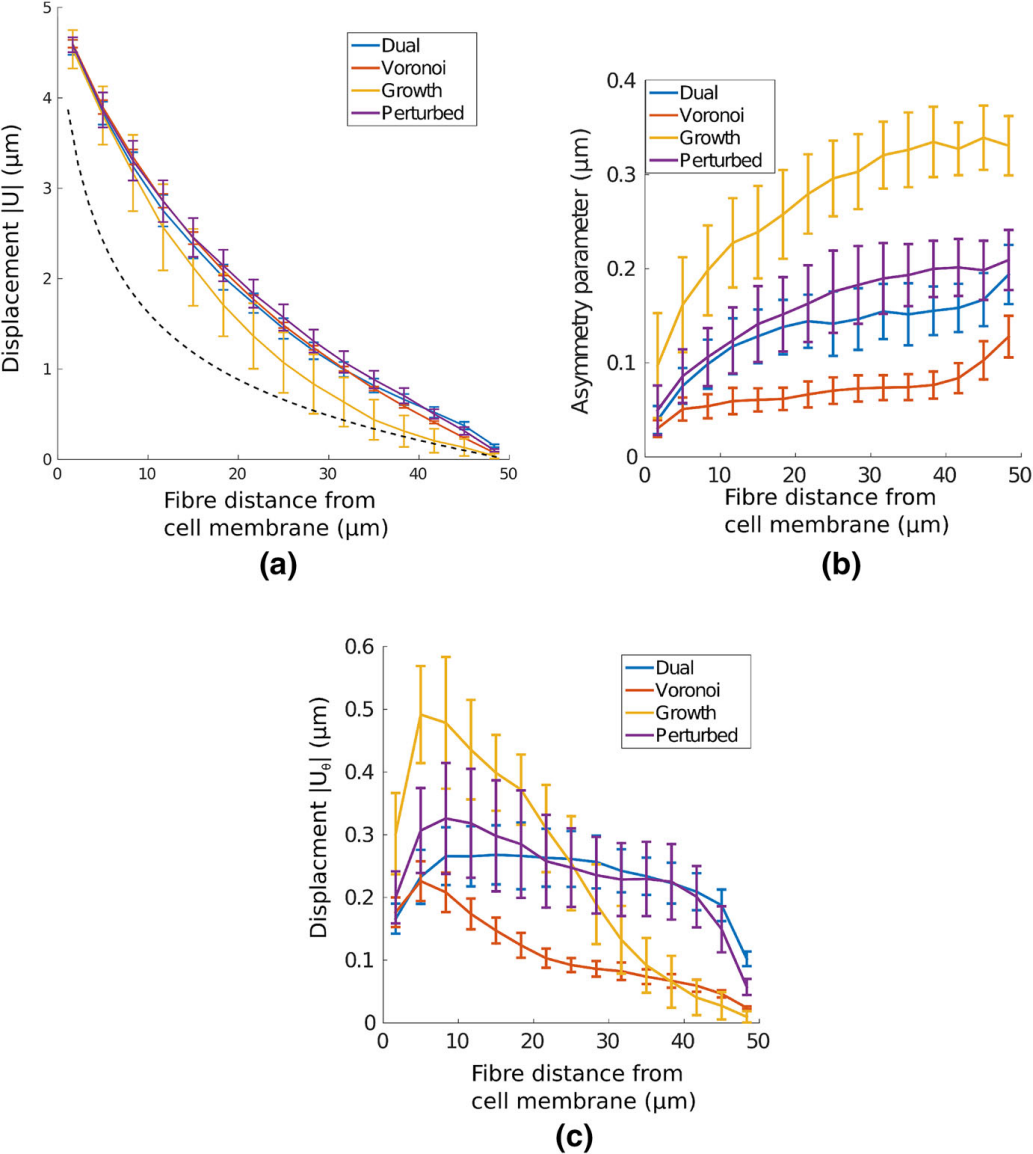
(Colour figure online) Comparison of displacement fields due to a contracting cell in different networks. **a** Mean displacement $|\text {U}|$ is plotted with distance from the cell membrane; long-range effects are observed for all cases considered, though growth networks extend considerably less far. In Voronoi, dual and perturbed networks, mean substrate displacement above ${1}\,\upmu \mathrm{m}$ is observed more than 3 (final) cell diameters distant. *Dashed line* shows equivalent displacement decay in a homogeneous elastic continuum. **b** Asymmetry in cell-derived displacement fields, showing the highly asymmetric fields in growth geometries, and the more symmetric fields induced within Voronoi networks. **c** Mean transverse displacement  as distance from the cell membrane increases. Significant transverse motion is present in growth networks, while displacement is largely radial in Voronoi cases. *Bars* give 1 SD about the mean value

Adherent cells contract and reorganise their substrates, leading to long-range displacement fields, extending many cell diameters (Winer et al. 2009). It is therefore important to quantify network response to local perturbations, and to investigate how far cell-derived substrate displacements reach. Interestingly, the choice of geometry profoundly affects cell-derived displacement fields. The magnitude of these displacement fields, given a uniformly contracting cell adhered to the substrate, is visualised in Fig. 4a–d. All networks supported a long-ranged displacement field, with significant substrate displacements (above ${1}\,\upmu \mathrm{m}$) between four and five cell diameters distant, similar to experimental results (Winer et al. 2009). The average mean displacement decay over 20 networks of each type is given in Fig. 5a, showing displacement fields reaching the outer boundary in dual, perturbed and Voronoi geometries. Remarkably, a similar mean displacement decay rate is observed in these three geometries, while growth networks supported a shorter-ranged mean field.

Inspection of the displacement fields arising in individual growth networks, as in Fig. 4c, shows that localised regions display network displacements of several micrometres many cell diameters away, while other regions are relatively unperturbed. As such, growth geometries support symmetry-breaking, which is also observed to a lesser extent in dual and perturbed networks. As a measure of asymmetry, each annular domain was binned into 12 angular segments. Mean displacement within each binned region was calculated, and normalised to the maximum value across all bins. The standard deviation in these quantities provides a measure of angular variation in the displacement fields, and therefore symmetry-breaking, as shown in Fig. 5b. While Voronoi networks yield approximately radially symmetric displacement fields, considerable angular variation is present in the other three geometries tested. In particular, growth type networks display high displacement field asymmetry.

**Fig. 6:**
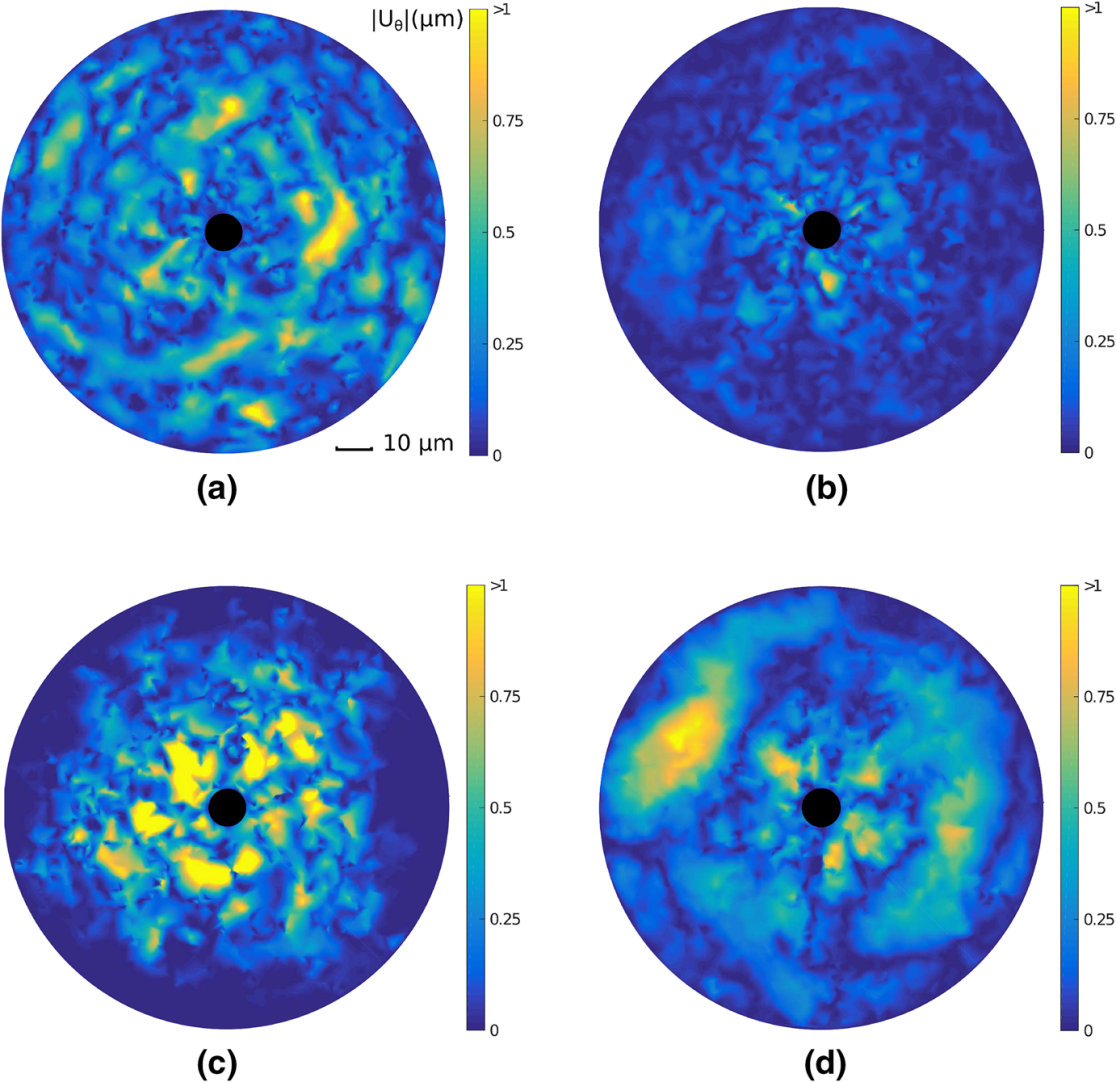
(Colour figure online) Visualisations of transverse displacement fields $|\text {U}_{\uptheta }|$ ($\upmu \mathrm{m}$) due to a single contractile cell in each network. Considerable transverse displacements, above ${1}\,\upmu \mathrm{m}$, are observed in growth networks a large distance from the cell membrane, while almost all displacement in Voronoi geometries is radial

Despite purely radial cell contraction, considerable transverse network displacements are observed. Visualisations of these displacement fields $|U_{\uptheta }|$ are shown in Fig. 6a–d, and quantified in Fig. 5c; except for in Voronoi cases, significant transverse motion is observed, particularly in growth networks.

The above results describe a uniform radial contraction within a nominally isotropic network inducing an asymmetric displacement field in certain geometries. This has important implications for cells communicating through substrate displacements (Reinhart-King et al. 2008), as the extent and direction of displacement is not robust in certain network geometries.

### Network Choice Strongly Influences Stress Transmission and Membrane Reaction Force

**Fig. 7:**
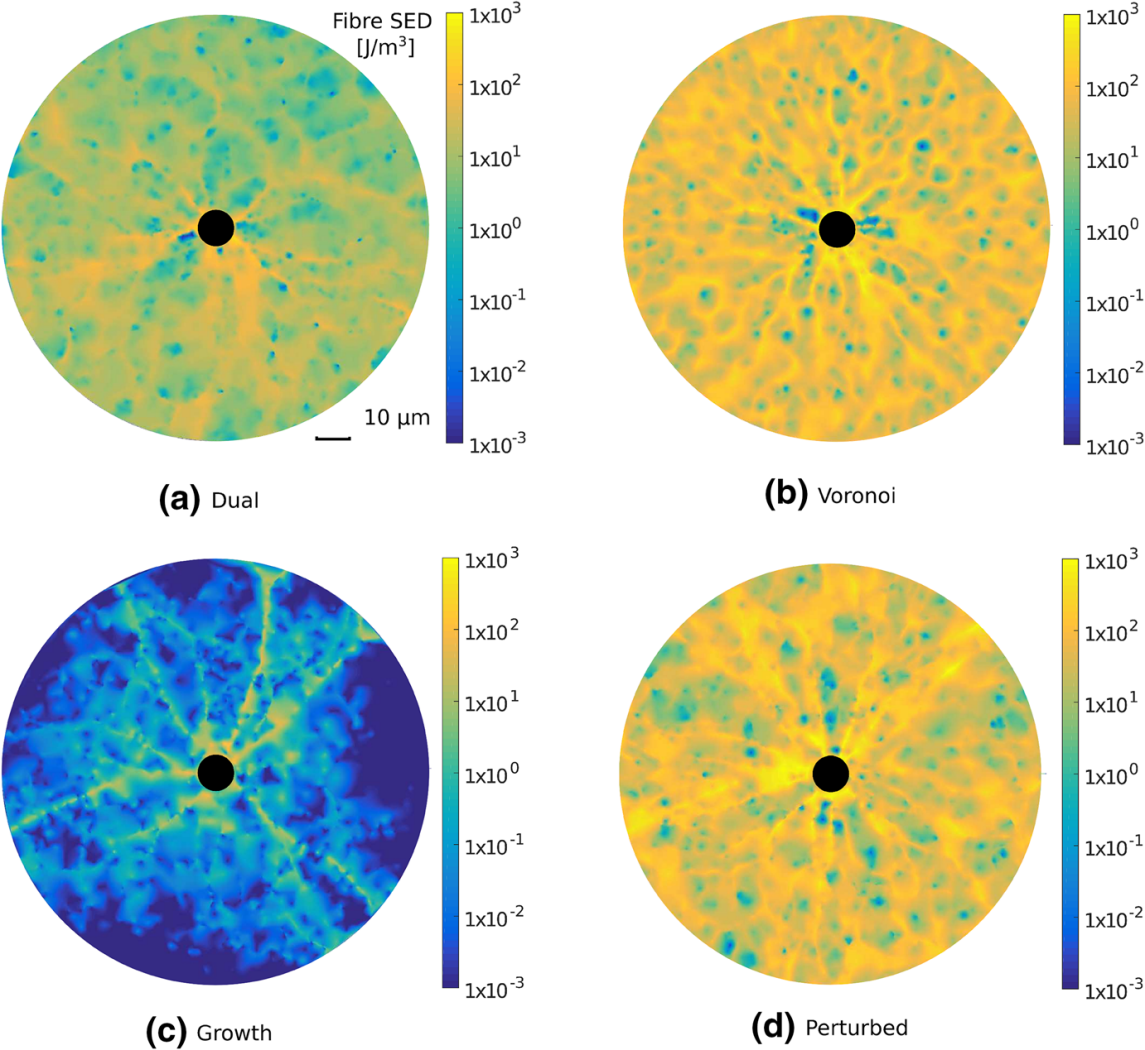
(Colour figure online) Strain energy density (SED) fields derived from single cells contracting in each network. Highly heterogeneous, shorter-ranged fields are observed in growth geometries, while long-range stress transmission to the distant boundary is observed in Voronoi and perturbed networks. Note the order of magnitude differences between architectures, as illustrated by log scale *colour bars*

**Fig. 8:**
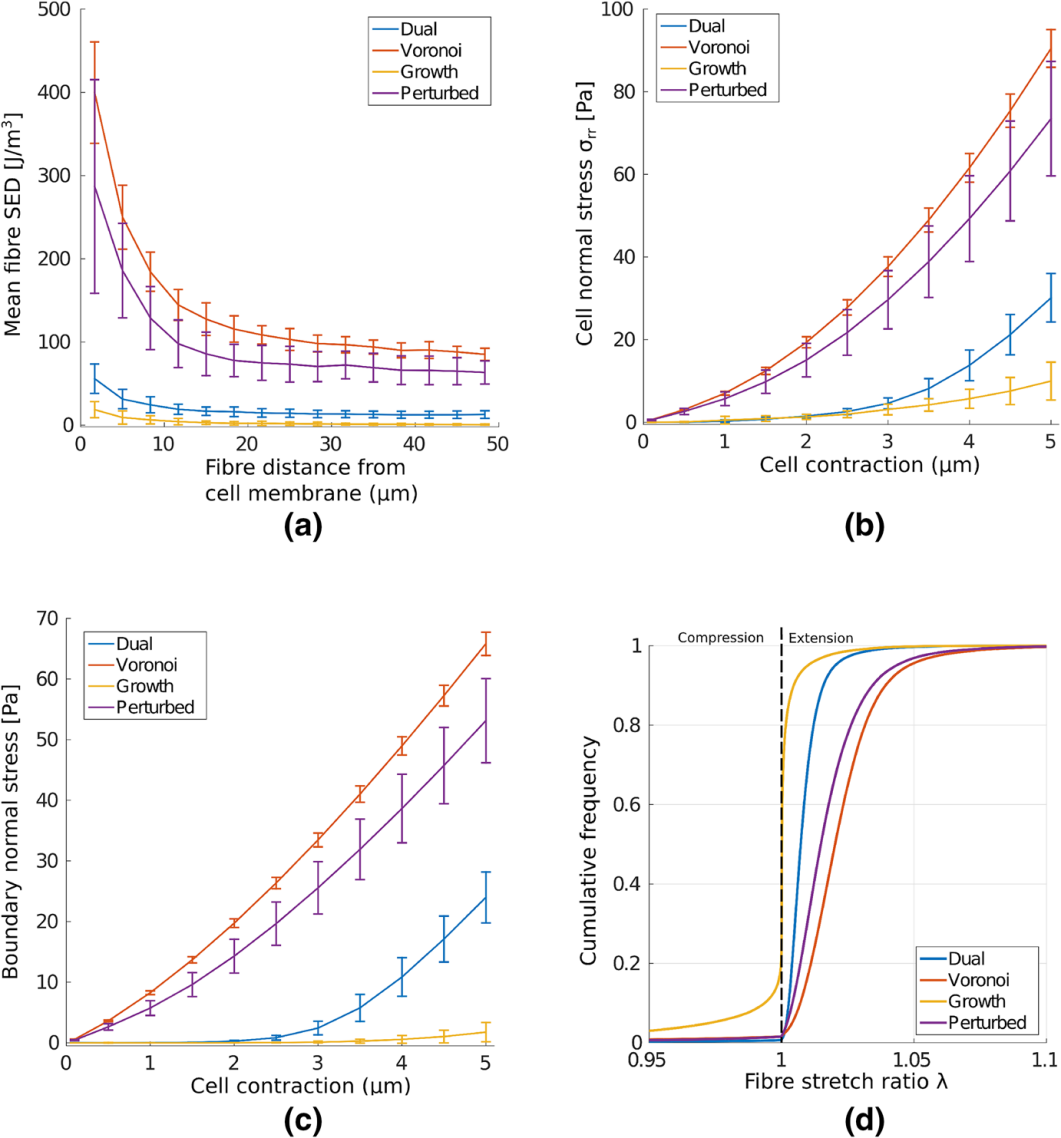
(Colour figure online) The transmission of stress in fibre networks. **a** Mean fibre strain energy density (SED) is plotted, showing far greater range of stress transmission in Voronoi and perturbed geometries than in growth or dual networks, with considerable strain energy more than 5 cell diameters distant. **b** Cells contracting in Voronoi and perturbed networks experience a far stiffer response than those in growth and dual networks. **c** Stress reaches the distant outer boundary in perturbed and Voronoi geometries, even at low cell contraction while in growth networks the distant boundary experiences very little stress. **d** Cell contraction leads to $20\%$ of growth network fibres buckling. The more shallow sigmoid found in Voronoi and perturbed geometries suggests that fibre strain is more equally distributed in these networks. *Bars* give 1 SD about the mean value

Next, we consider the nature of stress transmission through different network architectures. Cells contracting within compliant substrates transmit stress over long distances, allowing for the detection of distant cells and boundaries (Leong et al. 2010; Mohammadi et al. 2014). We investigate constituent fibre strains induced through the contraction of a single cell. To investigate stress transmitted through the substrate, individual fibre strain energy densities (SED) were calculated, according to:

$$\begin{aligned} \qquad \qquad W_{\text {f}} = \frac{1}{2} E \varepsilon ^2 \ , \end{aligned}$$

where $W_{\text {f}}$ is fibre SED, $\varepsilon = (l - L)/L$ is fibre strain, and *E* is the relevant Young’s modulus for extension or compression. Resulting fibre SED fields in each network geometry are visualised in Fig. 7, highlighting order of magnitude differences. In particular, growth geometries support limited stress transmission, in which localised families of fibres are strained and little stress is transmitted to the outer boundary. In contrast, Voronoi and perturbed networks support largely symmetric and far reaching fields. Stress transmission in dual networks is qualitatively similar to that in Voronoi and perturbed cases, though a greater degree of symmetry-breaking is observed, alongside a far lower SED. The decay of mean fibre SED with distance from the cell membrane is quantified in Fig. 8a, averaged over 20 network realisations. The decay of fibre SED with distance from the contracting cell is notably similar in perturbed and Voronoi networks, with SED decaying to a constant value far from the cell, suggesting the transmission of stress to the outer boundary. In contrast, significant strain is not transmitted beyond roughly one cell diameter distant in growth and dual cases. We note that despite a similar displacement decay rate for dual, Voronoi and perturbed architectures, significant differences arise in the ability to transmit stress through the network.

Cells respond sensitively to substrate stiffness, likely through a stress-limited mechanism (Winer et al. 2009; Discher et al. 2005). We quantify the stress at the cell membrane as a measure of how stiff a substrate appears to a contractile cell in Fig. 8b. In particular, we calculate normal membrane stress as the sum, over fibres connected to the cell membrane, of the normal components of fibre reaction forces, divided by the original cell membrane area. Despite equivalent cross-link density and material parameters, cells contracting in Voronoi and perturbed geometries experience a significantly stiffer response than those in growth or dual networks. The onset of strain-stiffening is observed in Voronoi and perturbed geometries at a contraction of approximately ${1.5}\,\upmu \mathrm{m}$, and to a lesser extent in dual networks at around ${3}\,\upmu \mathrm{m}$. The strain-stiffening response is notably weak in growth networks, which could have a profound effect on cell behaviour (Wen and Janmey 2013). The transmission of stress to the distant outer boundary is quantified in Fig. 8c. Cell contraction induces a reaction force at the outer boundary in Voronoi and perturbed geometries, even for small cell contractions. Within dual type networks, significant stress response at the outer boundary occurs once cell contraction reaches approximately ${2.5}\,\upmu \mathrm{m}$, leading to a clear stress signal at full contraction. In contrast, very little stress is transmitted to the boundary in growth networks, suggesting long-range detection of boundaries might not be possible in this geometry.

We investigate the fibre scale origins of these significant differences in strain and stress transmission. Cumulative fibre stretch distribution is given in Fig. 8d for each of the four networks considered. Importantly, approximately 20% of fibres in growth networks buckle or compress under cell contraction, with significant strain, above 1%, localised to just 10% of total fibre segments. Fibres within dual networks do not display the same extent of buckling, though have generally lower fibre stretch, with the steep sigmoid suggesting dominant contributions to strain energy are contained within relatively few fibres. In contrast, strain is distributed more evenly throughout perturbed and Voronoi geometries, with fewer buckled fibres, and 80–90% of fibres above 1% strain. Similarities between strain distributions in perturbed and Voronoi cases suggest that the perturbation applied to growth networks encourages a geometry which favours long-range stress transmission.

### Fibre Reorganisation Far from the Cell Membrane Depends on Geometry Choice, and Correlates with Fibre Strain

We now seek an explanation for the qualitative differences identified in the displacement and SED fields in contrasting network geometries. In particular, we investigate correlations between fibre stretch ratio and fibre alignment, and the rotation of constituent fibre segments with distance from the cell membrane.

**Fig. 9:**
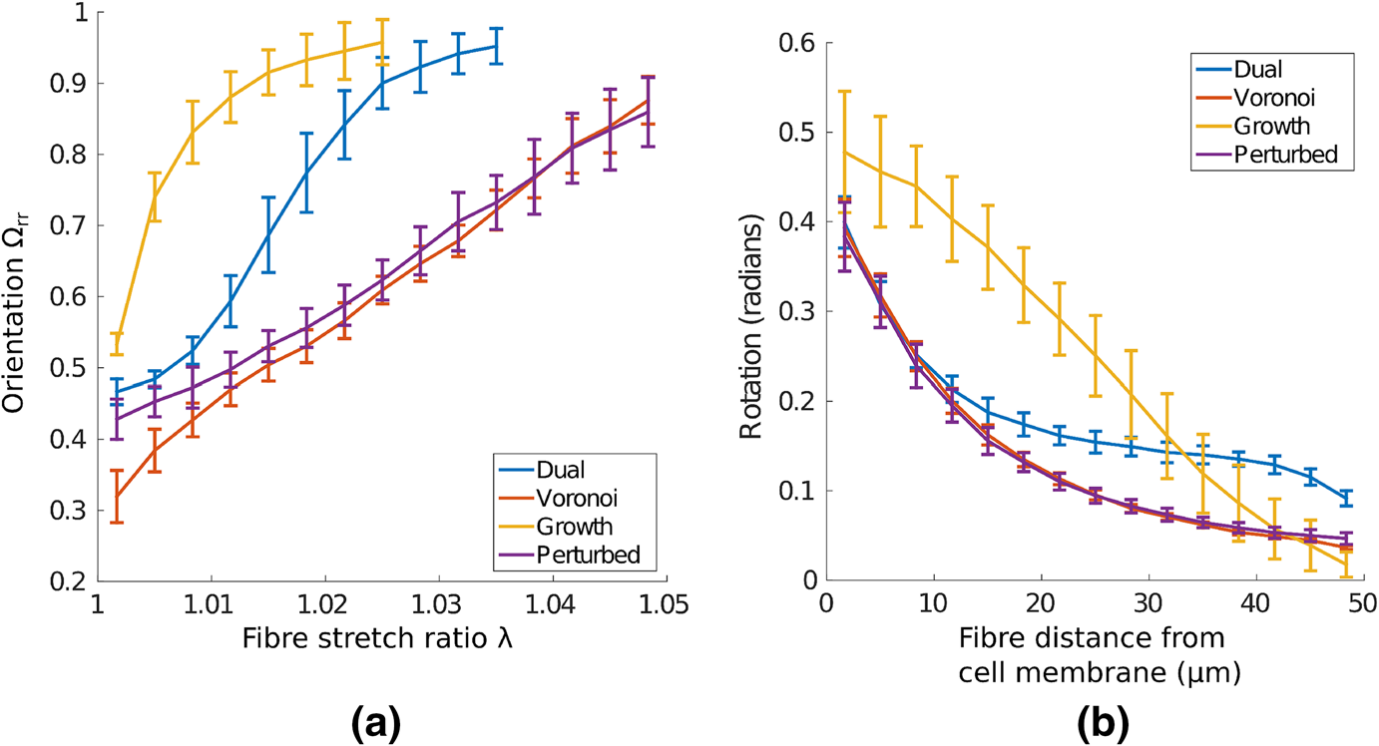
(Colour figure online) Alignment and reorganisation of non-buckled fibres. **a** Orientation of fibre families with increasing stretch ratio is plotted, showing a strong correlation between increasing fibre stretch and radial alignment in all networks. The strain distribution in Voronoi and perturbed geometries allows for families of fibres under significant strain (1–2$\%$) to remain unaligned with the direction of bulk stretch. **b** Mean rotation (radians) of constituent fibres within a network is plotted. Considerable fibre rotation is observed in growth networks, and far from the cell boundary in dual networks. The geometry of Voronoi and perturbed networks restricts rotation of fibres far from the cell membrane, and encourages stretching of individual network fibres. *Bars* give 1 SD about the mean value

The parameter $\Omega _\mathrm{rr}$, the radial analogue of Eq. (1), measures the radial alignment of fibres. Fig. 9 a shows the relation between this alignment parameter and fibre stretch. In all networks, increasing fibre stretch correlates with increased alignment. In growth networks, the most strained fibres are almost all highly aligned in the radial direction. This result is largely true in dual networks, though fibre strains are larger in general. However, the lower alignment value for $\Omega _\mathrm{rr}$ in perturbed and Voronoi cases suggests that the most strained fibres can exist in a variety of conformations, both aligned and unaligned. Interestingly, in both Voronoi and perturbed cases, we see that fibres up to 1% strain are more likely to exist in an unaligned configuration. As such, geometric differences in these networks allow for stretched fibres to exist in a greater range of configurations, allowing for enhanced stress transmission in comparison with growth and dual networks.

To investigate why fibres undergo higher stretch in some geometries, thus propagating stress, we quantify fibre rotation, that is the angle through which constituent fibres rotate, with distance from the cell membrane in Fig. 9b. Fibre segments in growth networks move through larger rotation arcs on average, implying that these network architectures allow for the reorganisation of fibres. The rotational behaviour of fibres in Voronoi and perturbed cases is very similar, with little rotation far from the cell boundary. Given that fibre SED extends far from the cell membrane, this implies that fibres are constrained to stretch rather than rotate, thus allowing for the greater extent of stress transmission, as seen in Fig. 7. Dual networks behave similarly to Voronoi and perturbed cases close to the cell membrane, though the higher degree of fibre rotation beyond two cell diameters away suggests that this geometry is not as constrained with regards to fibre reorganisation. It is interesting to note that a purely geometric difference, between growth and perturbed networks, encourages or discourages fibre stretch over rotation. These results offer a micromechanical explanation as to how geometry can promote qualitative differences in network response to local perturbations.

### The Dependence of Mechanical Response upon Network Geometry Persists at Higher Densities

**Fig. 10:**
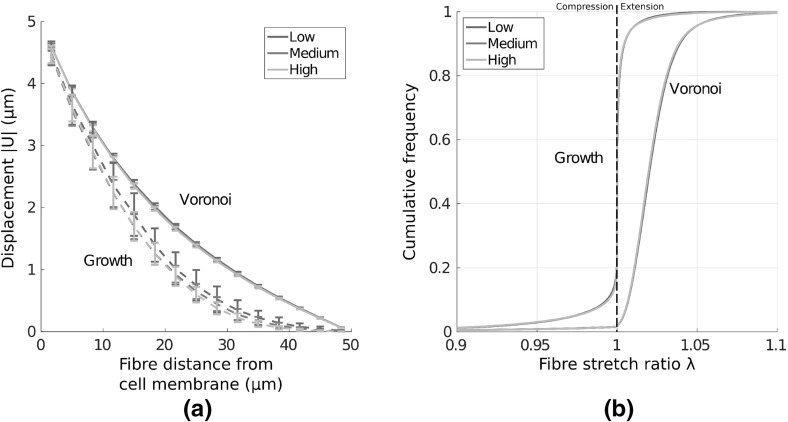
(Colour figure online) Important differences in network response persist for a range of densities. **a** Mean displacement $|\text {U}|$ is plotted with distance from the cell membrane, for three cross-link densities. A slight variation, within 1 SD, is found upon increasing fibre density. **b** Cumulative frequency plots for fibre stretch ratio is shown. The distributions do not vary significantly under a twofold increase in cross-link density. As such, fibre micromechanics are heavily dependent on network architecture for the range of densities investigated. *Bars* represent 1 SD about the mean value

Before comparing the ability for pairwise cells to mechanically communicate through networks of different geometries, we first investigate whether the differences in stress transmission and displacement fields depend strongly on network density. That is, we investigate whether the differences are robust to increases in density. As such, we compare displacement and fibre stretch results for networks of 1.5 and 2 times cross-link density, which corresponds to cross-link densities of 0.3 and ${0.4}\,\upmu {\mathrm{m}}^2$ respectively. As network density increases, inter-cross-link length reduces and we might expect networks to approach an affine limit. Displacement with distance from the cell membrane for Voronoi and growth networks of the three densities considered are shown in Fig. 10a. We find that variation in results is slight, and clearly within one standard deviation for individual networks of equal density, suggesting the extent of cell-derived displacement fields persists at a range of relevant densities. Further, cumulative fibre stretch distributions shows no discernible differences as network density increases, as shown in Fig. 10b. As such, important differences persist as network density is increased. This insensitivity to a twofold increase in cross-link density suggests that differences arising from network architecture are relevant on a range of scales, and allows for the investigation of pairwise interactions at a single density without loss of generality.

### Pairwise Contractile Cells can Mechanically Signal Through Transmitted Stress in Certain Architectures

**Fig. 11:**
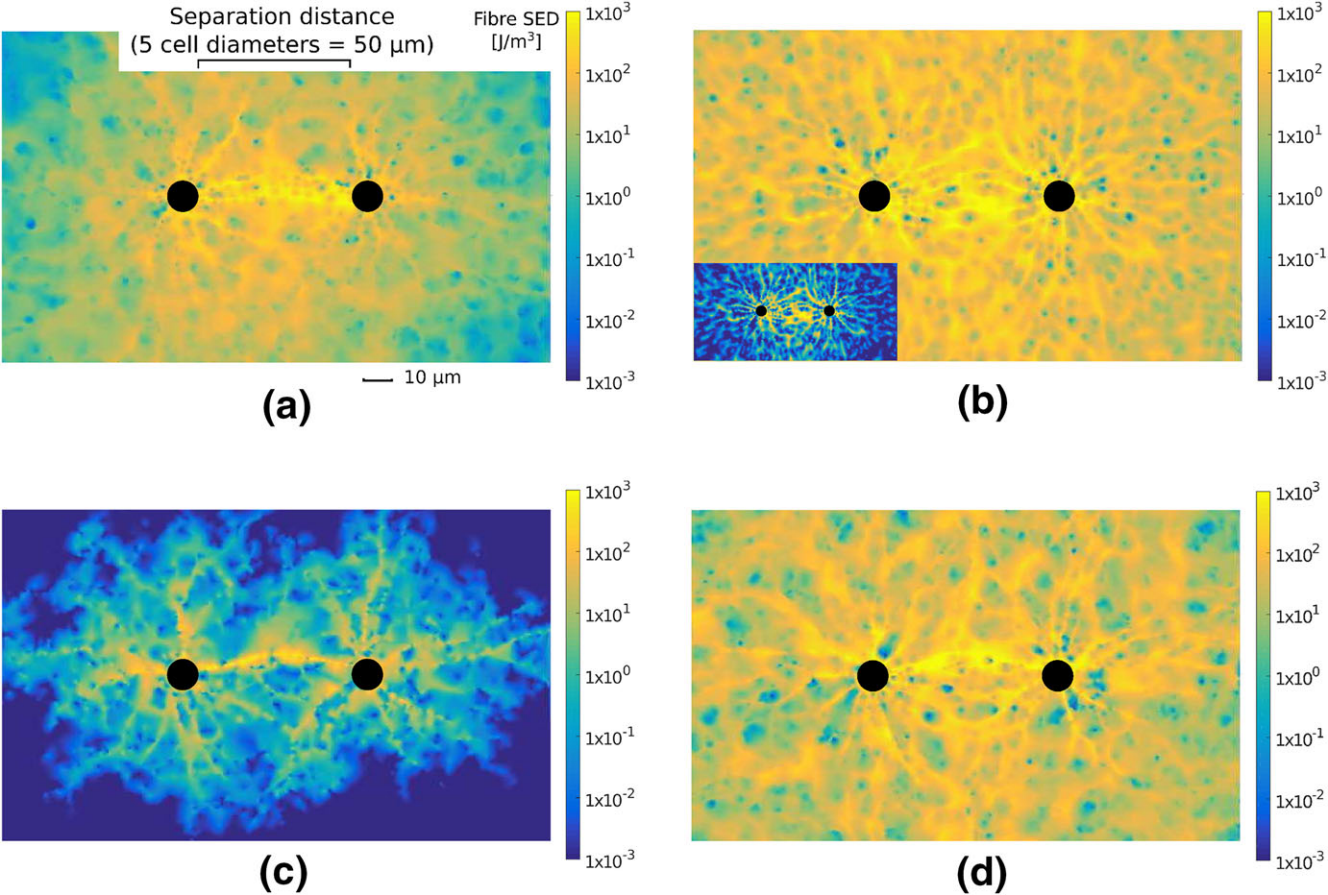
(Colour figure online) Visualisations of fibre strain energy density for pairwise contracting cells at a separation distance of ${50}\,\upmu \mathrm{m}=$ 5 final cell diameters. Increased strain energy density in the intercellular region is clear in all networks, as evidence by the bright band in between cells. Note the log scale *colour bars*, underlining order of magnitude differences in strain energy fields. **b** (*Inset*) to highlight the presence of SED banding between cells in the Voronoi case, a rescaled plot is included. Logarithmic *colour* scaling gives one order of magnitude difference. **a** Dual, **b** Voronoi, **c** growth, **d** perturbed

**Fig. 12:**
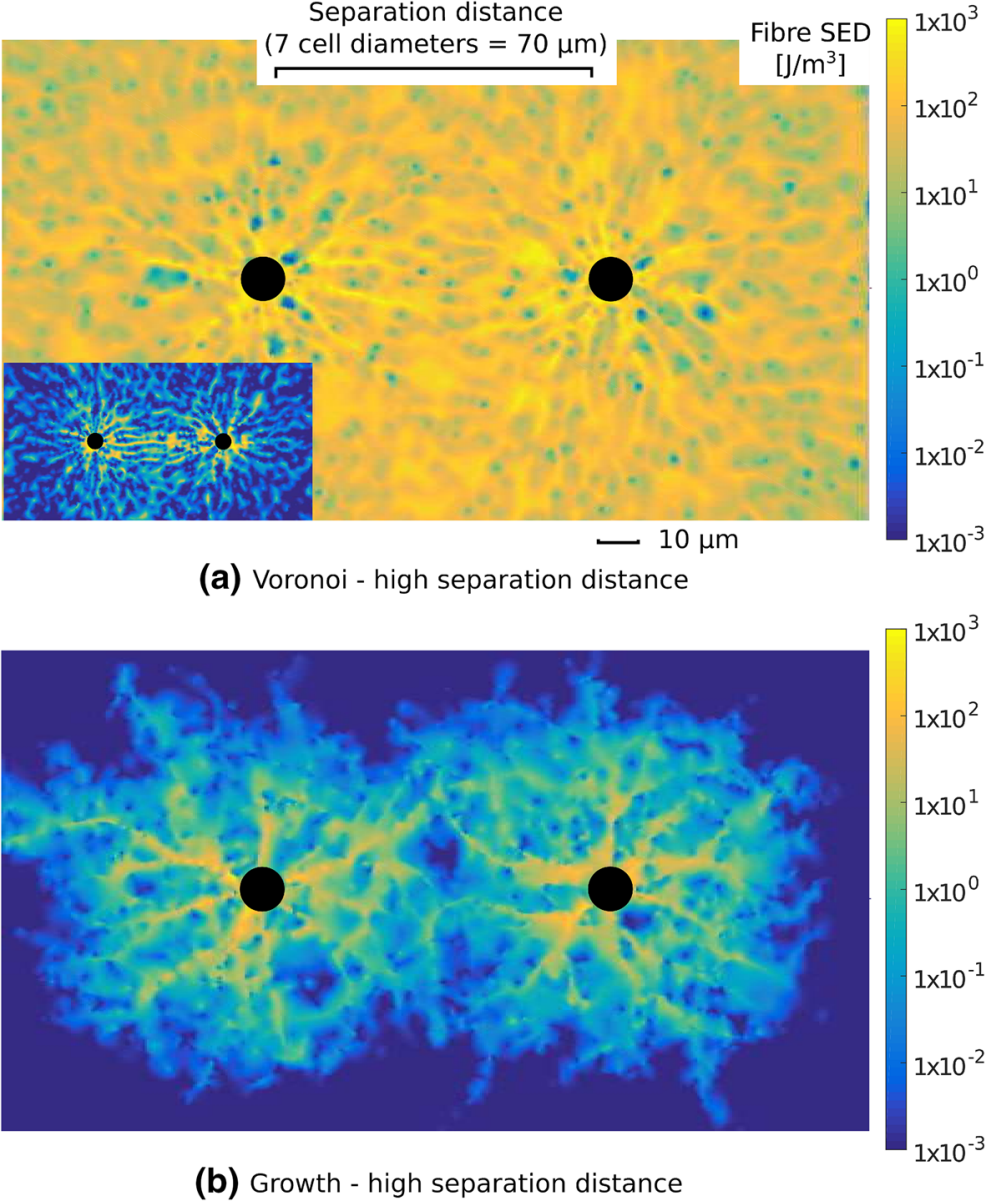
(Colour figure online) Visualisations of fibre strain energy density for cells contracting at maximum separation distance ${70}\,\upmu \mathrm{m} =$ 7 cell diameters. **a** Increased strain energy between cells is observed for cells contracting in Voronoi networks at a higher separation distance of 7 cell diameters. *Inset* is a rescaled plot to highlight SED banding between cells. Logarithmic *colour* scaling gives one order of magnitude difference in this case. **b** No such increase is found in growth networks, suggesting that cells cannot robustly communicate mechanically over such distances in this architecture

**Fig. 13:**
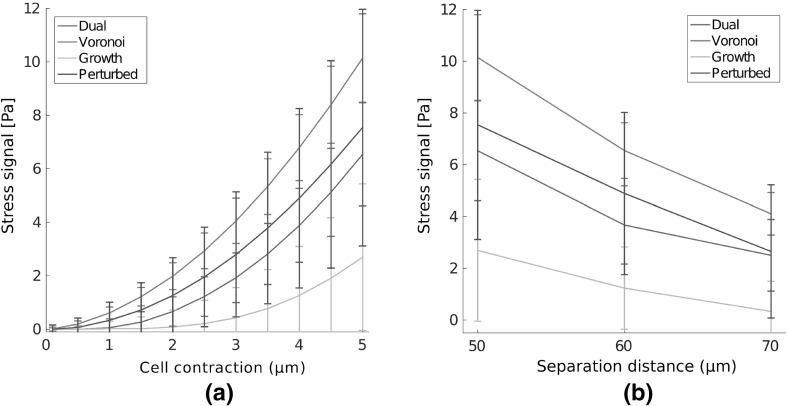
(Colour figure online) Mechanical communication via stress transmission through different network architectures. **a** The stress signal at the cell membranes as cell contraction increases is plotted for separation distance of ${50}\,\upmu \mathrm{m}$ = 5 final cell diameters. A robust, stronger signal is observed in Voronoi networks, with weaker, variable signal in growth geometries. Large standard deviation in perturbed cases underlines high network-to-network variability in signal propagation. **b** The stress signal at maximal contraction (${5}\,\upmu \mathrm{m}$) for increasing cell separation is plotted. Significant signal is transmitted between cells which are 7 cell diameters distant in Voronoi networks, while robust stress transmission beyond 5 cell diameters of separation fails in growth networks. *Bars* give 1 SD about the mean value

Cells possess the ability to communicate mechanically through substrates, with pairwise cell motility influenced by substrate compliance (Reinhart-King et al. 2008), and alignment of both cells and substrate over long distances (Winer et al. 2009). We investigate the ability for mechanical signals to be transmitted between pairs of cells through different network geometries, as shown schematically in Fig. 2c. Fibre SED within networks containing pairs of cells at three distances, corresponding to separations of 5, 6 and 7 (final) cell diameters, is investigated. Single network visualisations are shown in Fig. 11 for cell separations of five cell diameters. In this case, where the separation distance is relatively small, clear bands of high strain energy are visible between contracting cells. At greater separations, corresponding to 7 cell diameters, as shown in Fig. 12a–b, increased SED between cells is still clear in Voronoi networks (a), while this banding is minimal or absent in growth geometries (b). We define a mechanical signal between the two cells to be the difference in normal stress between the two halves of the cell, that is the halves facing towards and away from the other cell. In particular, if the cell membrane stress on the half facing towards a distant cell is greater than that on the other half, this signal is positive. For a separation distance of 5 cell diameters, this signal with increasing cell contraction is shown in Fig. 13a. A clear stress signal at maximum radial contraction (${5}\,\upmu \mathrm{m}$) is observed across all geometries. However, the larger standard deviation in this signal for the growth network cases highlights the possibility that cells in these geometries fail to communicate mechanically. In contrast, the stronger signal, and smaller standard deviation, in Voronoi networks implies that there exists robust transmission of stress signal between cells.

Figure 13b shows the signal parameter in each network geometry for maximum radial contraction (${5}\,\upmu \mathrm{m}$) as separation distance is increased. Robust signalling persists in Voronoi networks, even at a larger separation of 7 cell diameters. A smaller signal is observed in both dual and perturbed networks on average, while growth geometries do not support a meaningful signal at high separation distance. Further, the large standard deviations for growth cases at all separation distances underline the possible failure of cells to achieve a signal. In all cases, the most robust (smallest standard deviation) and largest mean signals derive from Voronoi network geometries. Interestingly, the change in geometry type, from growth to perturbed, yields two networks whose ability to support mechanical signalling, as defined above, is entirely different. Perturbed networks support signals of four times the amplitude found in growth cases at the shortest distance considered, and with a stronger signal at 7 cell diameter separation than found in growth networks at 5 cell diameter separation, emphasising that stress transmission can be sensitive to network geometry.

## Conclusions

We have presented a whole network model for fibrous substrates which employed four different geometries. These architectures were chosen to investigate the importance of geometry for network mechanical response in a variety of biologically relevant cases. The dominant effect of network geometry was investigated by controlling for topology, with each network paired with another of highly similar or identical topology. All networks considered were nominally isotropic at the whole network scale, as verified by the Kolmogorov–Smirnov test. Through a simple model for constituent fibres and cross-links, we demonstrated that purely geometric differences can have a profound effect on network mechanics. These effects were investigated for networks under uniaxial extension, as well as for systems of one and two cells contracting within a network. No two network geometries exhibited similar results under all computational tests performed, and these results were found to be robust to a factor two change in network cross-link density. The primary conclusions from this work may be described as follows.

### Qualitative Differences in Long-Ranged Displacement Fields Due to Single Contracting Cells

All networks supported long-ranged displacement fields, with significant substrate displacement beyond four cell diameters. However, growth type geometries had a considerably shorter mean displacement field, and substantial displacements were localised to branching regions from the cell membrane. Significant asymmetry was observed for growth geometries, and to a lesser extent for dual and perturbed networks, though Voronoi architectures supported largely symmetric displacement fields. Significant transverse network displacements that were greater than ${1}\,\upmu \mathrm{m}$ in magnitude arose from uniform radial cell contraction in growth networks. As such, we found that certain geometries supported heterogeneous, asymmetric displacement fields arising under uniform contraction in isotropic networks. Importantly, the geometric perturbation of growth networks substantially increased the extent of cell-derived mean displacement fields, and reduced the degree of asymmetry.

### Stress Transmission Through Fibrous Networks is Dependent on Network Choice

The degree to which stress could be transmitted through fibre networks was found to be heavily dependent on geometry. In central force networks stress is transmitted through constituent fibre strain. In Voronoi and perturbed geometries, cell-derived network deformations transmitted stress to boundaries more than 5 cell diameters distant, even at low contraction. In these architectures, cells experienced greater membrane stress, with strain-stiffening response for increasing contraction. In contrast, little stress was transmitted to the distant outer boundary in growth or dual networks. Cells experienced these networks to be softer, with minimal strain-stiffening in growth networks. As such, the perturbation performed to growth networks fundamentally changed phenomena characterised in the context of local deformations. Interestingly, fibre strain distributions showed that many more fibres buckled in growth networks, suggesting that the softer response may be due to a geometry encouraging fibre buckling.

### Choice of Geometry can Favour or Limit Fibre Reorganisation

High radial alignment of fibres was found in the pericellular region, within 1 cell diameter of the cell membrane, in all network choices. However, the extent of fibre reorganisation with increasing distance from the cell membrane depended heavily on network architecture. Voronoi and perturbed geometries limited the degree to which fibres far from the cell membrane could rotate. This allowed for families of stretched, but unaligned fibres to exist within a network, amplifying stress transmission. In contrast, growth and dual networks allowed for a greater rotation of distant fibres, so that strains induced by cell contractions could be contained in fibre reorganisation rather than fibre stretch. Strong correlation between fibre stretch and alignment was identified in all network architectures.

### Mechanical Communication Between Distant Cells is Geometry Dependent

Distant model cells, separated by 5 (final) cell diameters, were able to transmit a mechanical signal through fibre networks, with regions of higher strain energy banding in between cells. However, as separation distance increased, up to seven cell diameters, a meaningful signal was not transmitted in growth networks. At all separation distances investigated, more significant and robust signalling was present in Voronoi networks, underlining the possibility of long-range cell–cell mechanical communication in this architecture. Weaker signalling on average, compared to the Voronoi case, was present in dual and perturbed networks, with the latter exhibiting larger standard deviations highlighting the less robust response. Importantly, in growth networks signal transmission was minimal or absent at 6–7 cell diameter separation; in contrast their perturbed counterparts, possessing identical network topology, supported significantly stronger signals, suggesting a key role for geometry in mechanical cell–cell communication.

## Discussion

The above results provide an insight into the potential importance of network geometry to cell behaviour. Throughout, specific fibre material properties were not discussed, so as to facilitate a comparative study between different geometries. We might expect that stiffer filaments, such as semiflexible polymers characterised by long persistence lengths, would favour geometries in which correlations of fibre orientations are maintained through model cross-links. While this was the case in growth networks, such correlations were, in general, lost through nodes in the other architectures discussed, as seen in Fig. 1. However, different geometries are achievable in semiflexible polymer networks through variation of gelation temperatures. Indeed, collagen gels formed at lower temperatures exhibit more fibrous architectures, compared with those polymerised at higher temperatures, which appear more homogeneous even after subsequent cooling. Further, many biopolymers exhibit fibre branching, where filament bifurcations lead to network nodes with coordination three. As such, the results presented above are likely relevant to a wide range of substrate gels, though further work should investigate how different geometries might relate to in vitro networks.

We found that key aspects of a network’s response, such as the distribution of fibre strains, persisted even as cross-link density increased, and therefore fibre length decreased. While it is not obvious exactly why network response varies so drastically with changing network type, we note that there is a greater variation in segment lengths in growth networks, as compared to other geometries, along with a slightly longer mean segment length. As we move towards generating biopolymer networks with tunable cross-linking, the competing effects of mesh size, and cross-link and fibre densities upon network mechanics will be characterised. In the case of 3D networks, we expect that the ratio of fibre to cross-link density will be greater than in the 2D case, suggesting that growth networks would be a more prudent choice, while Voronoi geometries might better represent planar networks.

We simulated fibre microbuckling through the use of an asymmetric force-extension law, and freely rotating cross-links. Recent work suggests that it is this anisotropy that allows for mechanosensing in extracellular matrices (Notbohm et al. 2015). Prior work has suggested that bending deformations are important for characterising the response of fibre networks in certain regimes (Head et al. 2003a). However, even when bending dominates stretching energy, the resulting forces often still act in the axial direction, suggesting that fibre buckling behaviour is most important, rather than transverse deflections (Heussinger and Frey 2007). As such, we expect that the asymmetric force-extension law used captures the essential behaviour of the slender matrix fibres that they are far stiffer under extension than compression. However, it is important to note that while the asymmetric force-extension law might be the most important factor for cell–cell communication, the lack of a fibre bending modulus does imply that the networks lack a classical linear regime. As such, there exists a geometry-specific separation distance between two cells such that the displacement enforced by the model cells leads only to fibre rotation, without any segment strain, and therefore energy cost. In these situations of greater cell separation, or lesser cell-derived displacement, it would be more appropriate to model the constituent biopolymers as beams, or to introduce rigid cross-links enforcing bond angles. While these issues have not been explicitly addressed within this work, the differences identified under a range of mechanical tests suggest that network geometry is likely to play a key role in the mechanics of biological networks.
